# Supplementary material for: Gut Microbial Composition Differs Extensively among Indian Native Chicken Breeds Originated in Different Geographical Locations and a Commercial Broiler Line, but Breed-Specific, as Well as Across-Breed Core Microbiomes, Are Found
Source: Microorganisms. 2021 Feb 14;9(2):391. doi: 10.3390/microorganisms9020391 (PMC7918296; doi:10.3390/microorganisms9020391)
Supplement: Supplementary file 1 [file microorganisms-09-00391-s001.zip › Figure S1.pptx]

## Slide 1
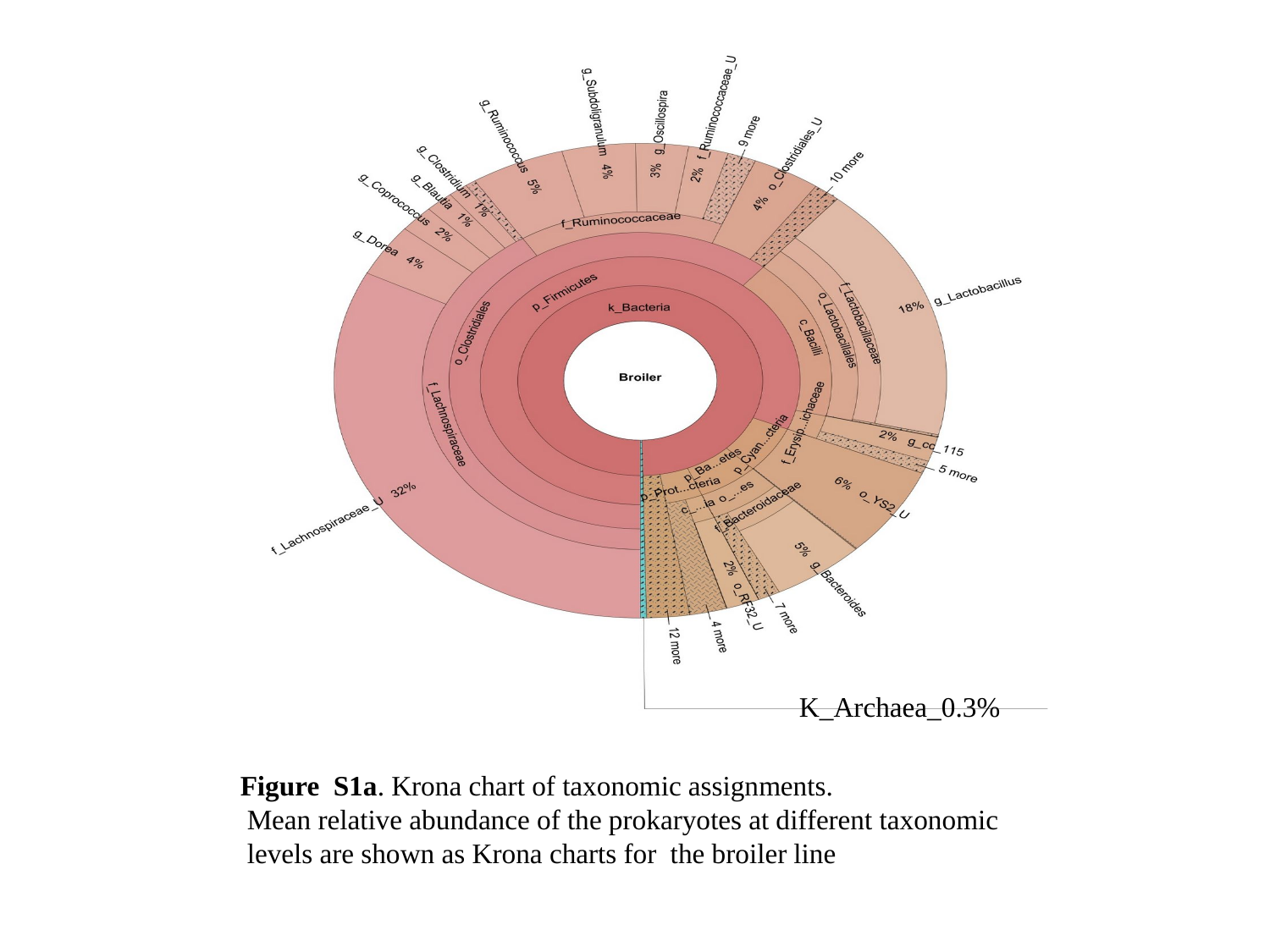

K_Archaea_0.3%
Figure S1a. Krona chart of taxonomic assignments.
 Mean relative abundance of the prokaryotes at different taxonomic
 levels are shown as Krona charts for the broiler line

## Slide 2
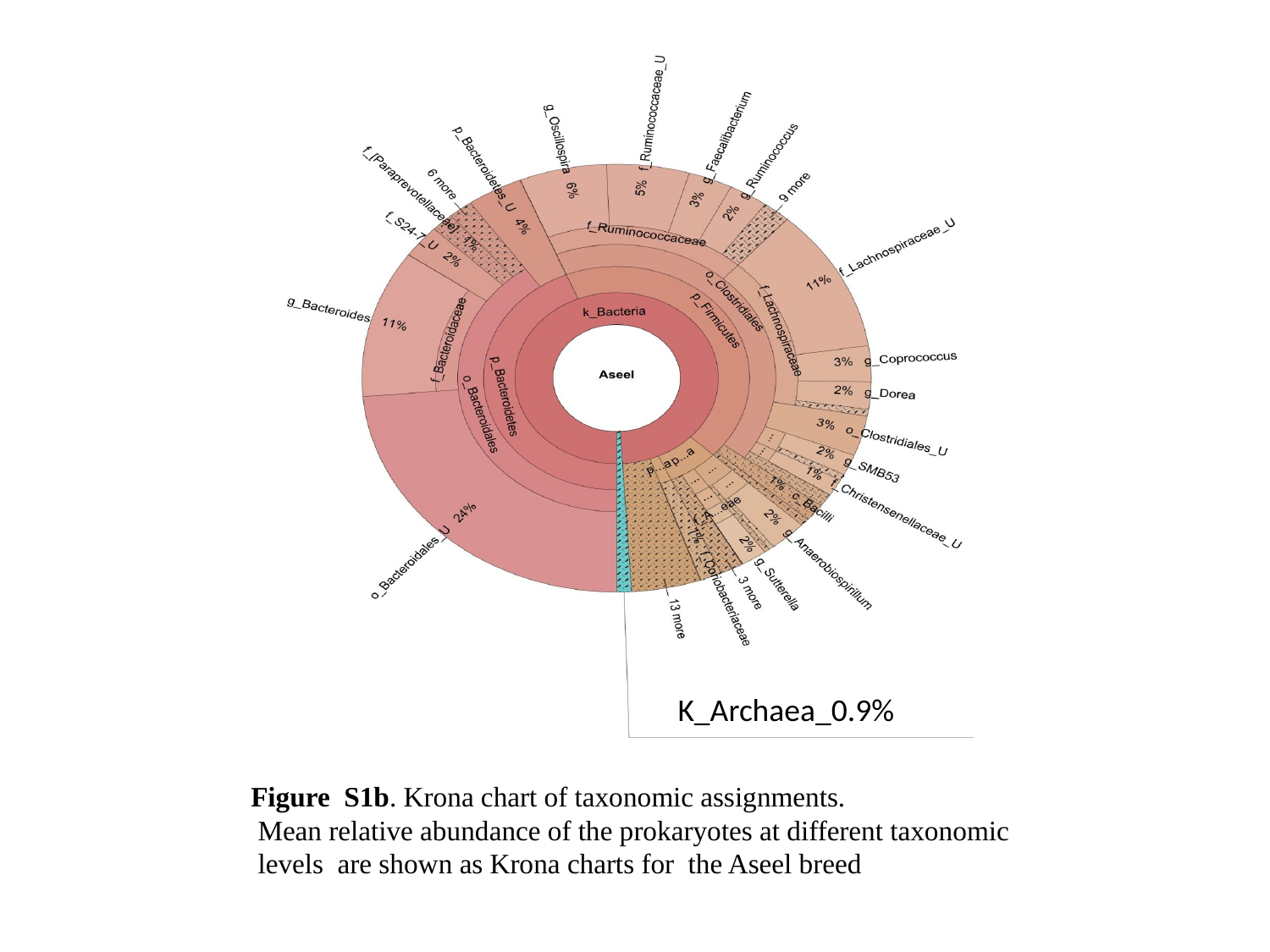

K_Archaea_0.9%
Figure S1b. Krona chart of taxonomic assignments.
 Mean relative abundance of the prokaryotes at different taxonomic
 levels are shown as Krona charts for the Aseel breed

## Slide 3
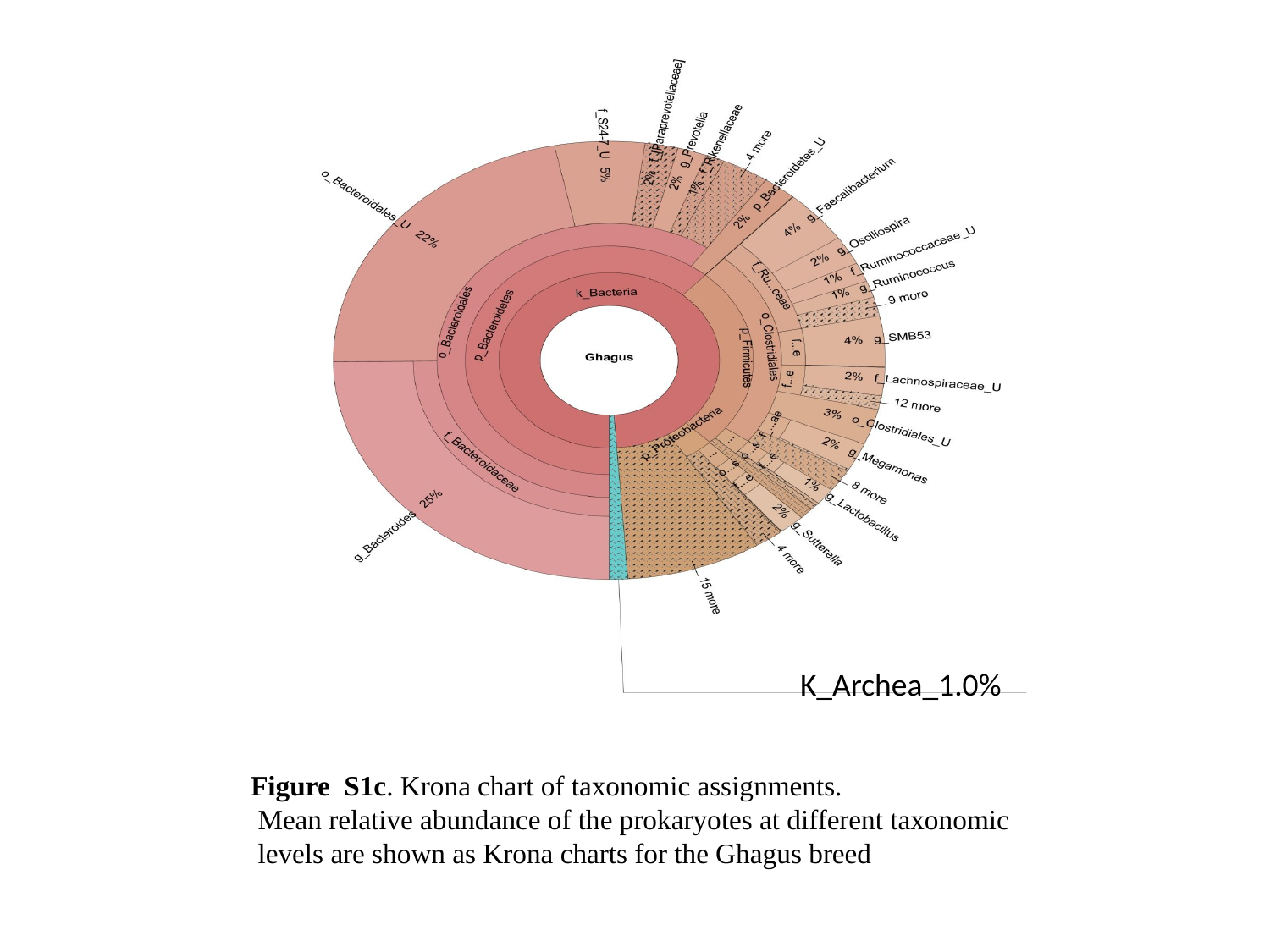

K_Archea_1.0%
Figure S1c. Krona chart of taxonomic assignments.
 Mean relative abundance of the prokaryotes at different taxonomic
 levels are shown as Krona charts for the Ghagus breed

## Slide 4
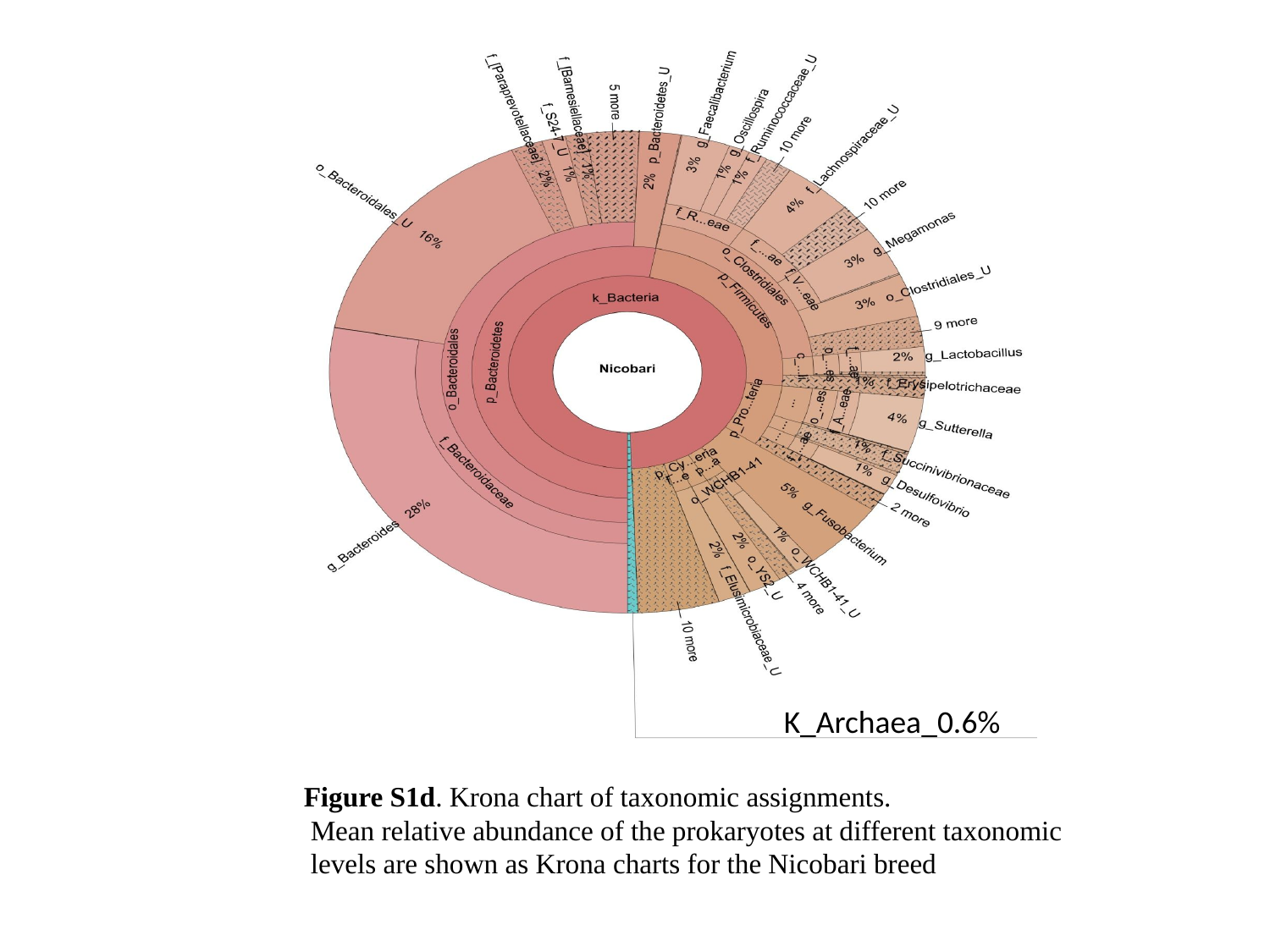

K_Archaea_0.6%
Figure S1d. Krona chart of taxonomic assignments.
 Mean relative abundance of the prokaryotes at different taxonomic
 levels are shown as Krona charts for the Nicobari breed

## Slide 5
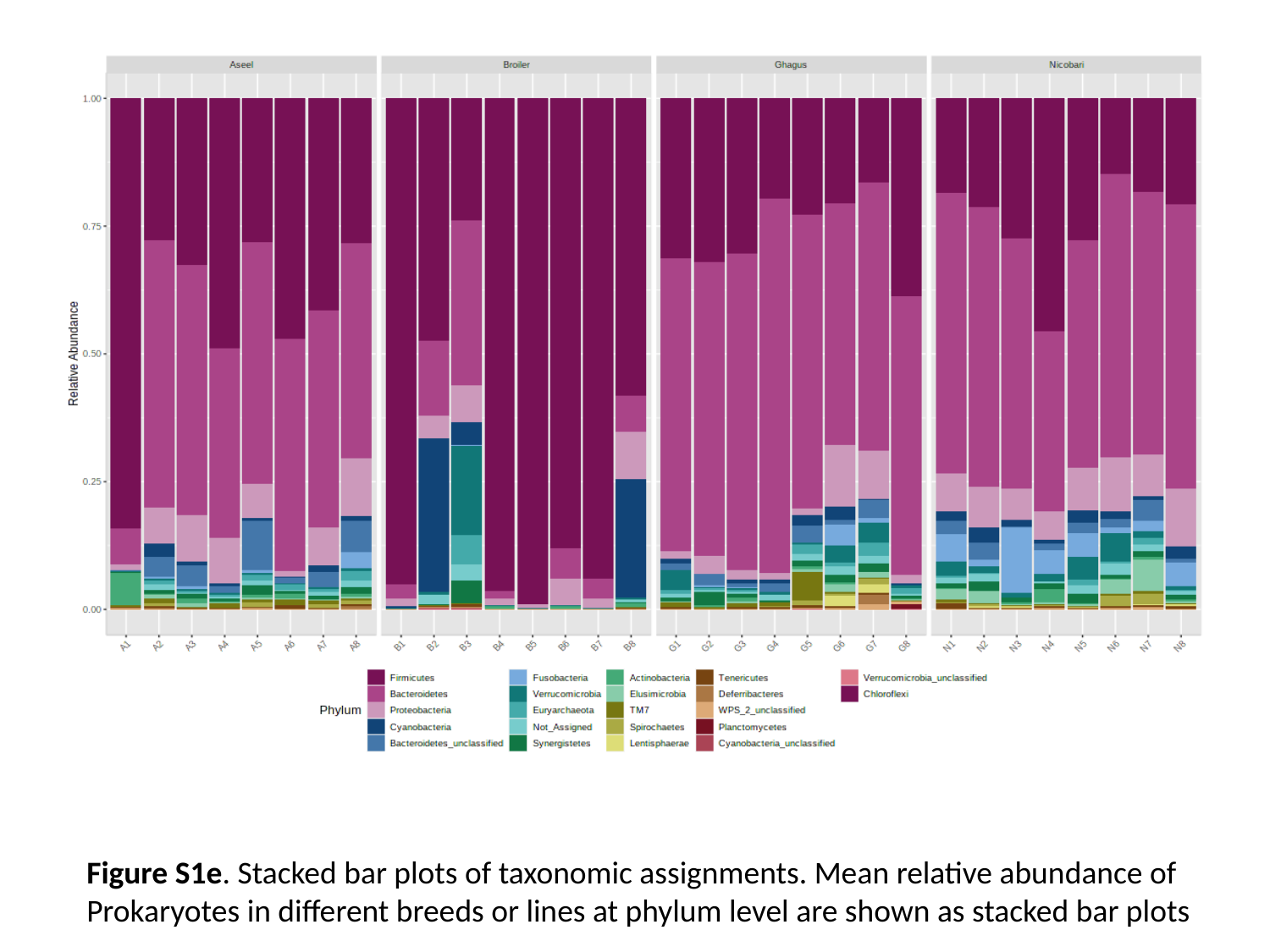

Figure S1e. Stacked bar plots of taxonomic assignments. Mean relative abundance of
Prokaryotes in different breeds or lines at phylum level are shown as stacked bar plots
