# Supplementary material for: Gut Microbial Composition Differs Extensively among Indian Native Chicken Breeds Originated in Different Geographical Locations and a Commercial Broiler Line, but Breed-Specific, as Well as Across-Breed Core Microbiomes, Are Found
Source: Microorganisms. 2021 Feb 14;9(2):391. doi: 10.3390/microorganisms9020391 (PMC7918296; doi:10.3390/microorganisms9020391)
Supplement: Supplementary file 1 [file microorganisms-09-00391-s001.zip › Figure S2.pptx]

## Slide 1
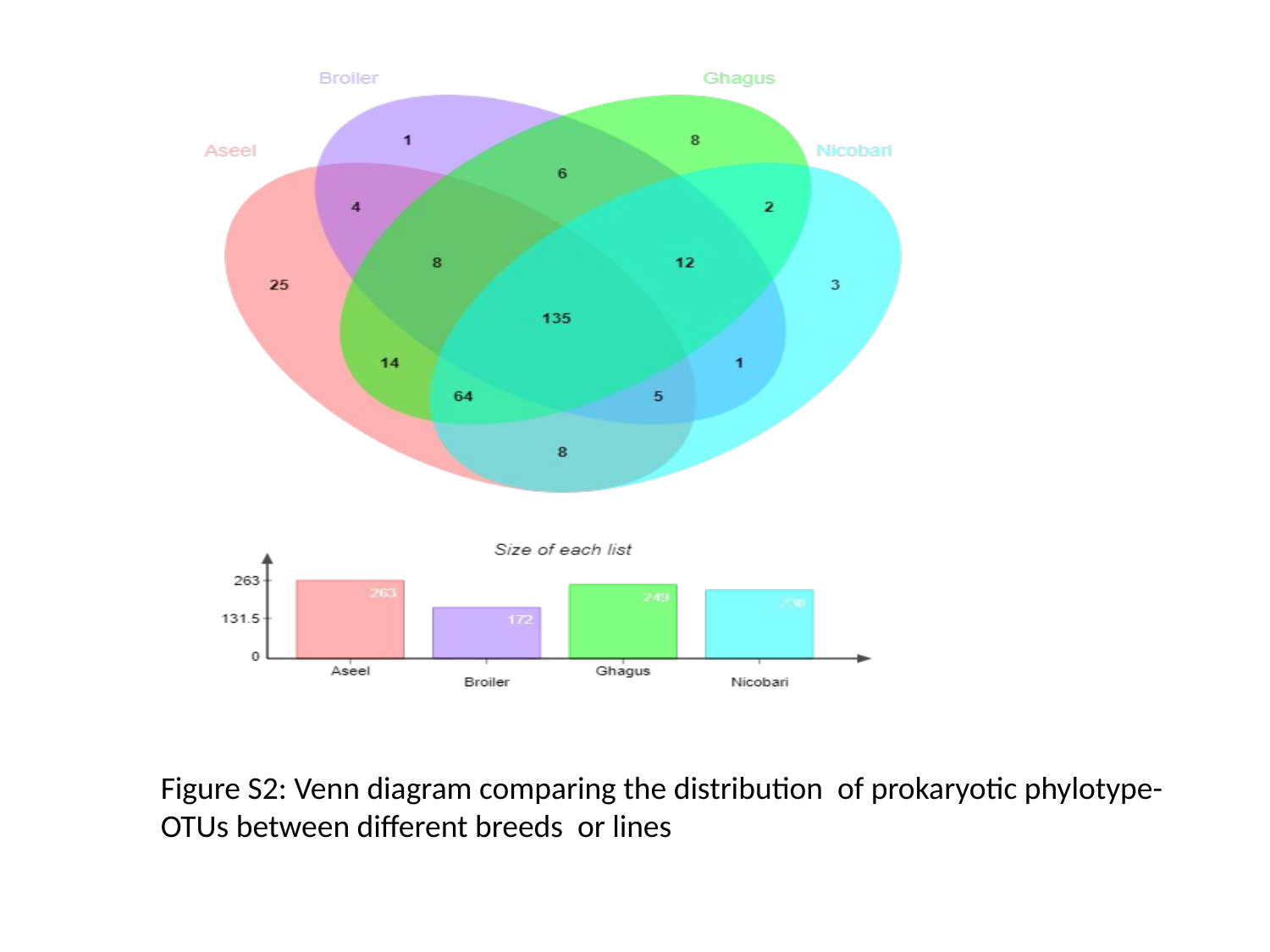

Figure S2: Venn diagram comparing the distribution of prokaryotic phylotype-OTUs between different breeds or lines
