# Supplementary material for: Gut Microbial Composition Differs Extensively among Indian Native Chicken Breeds Originated in Different Geographical Locations and a Commercial Broiler Line, but Breed-Specific, as Well as Across-Breed Core Microbiomes, Are Found
Source: Microorganisms. 2021 Feb 14;9(2):391. doi: 10.3390/microorganisms9020391 (PMC7918296; doi:10.3390/microorganisms9020391)
Supplement: Supplementary file 1 [file microorganisms-09-00391-s001.zip › Figure S3.pptx]

## Slide 1
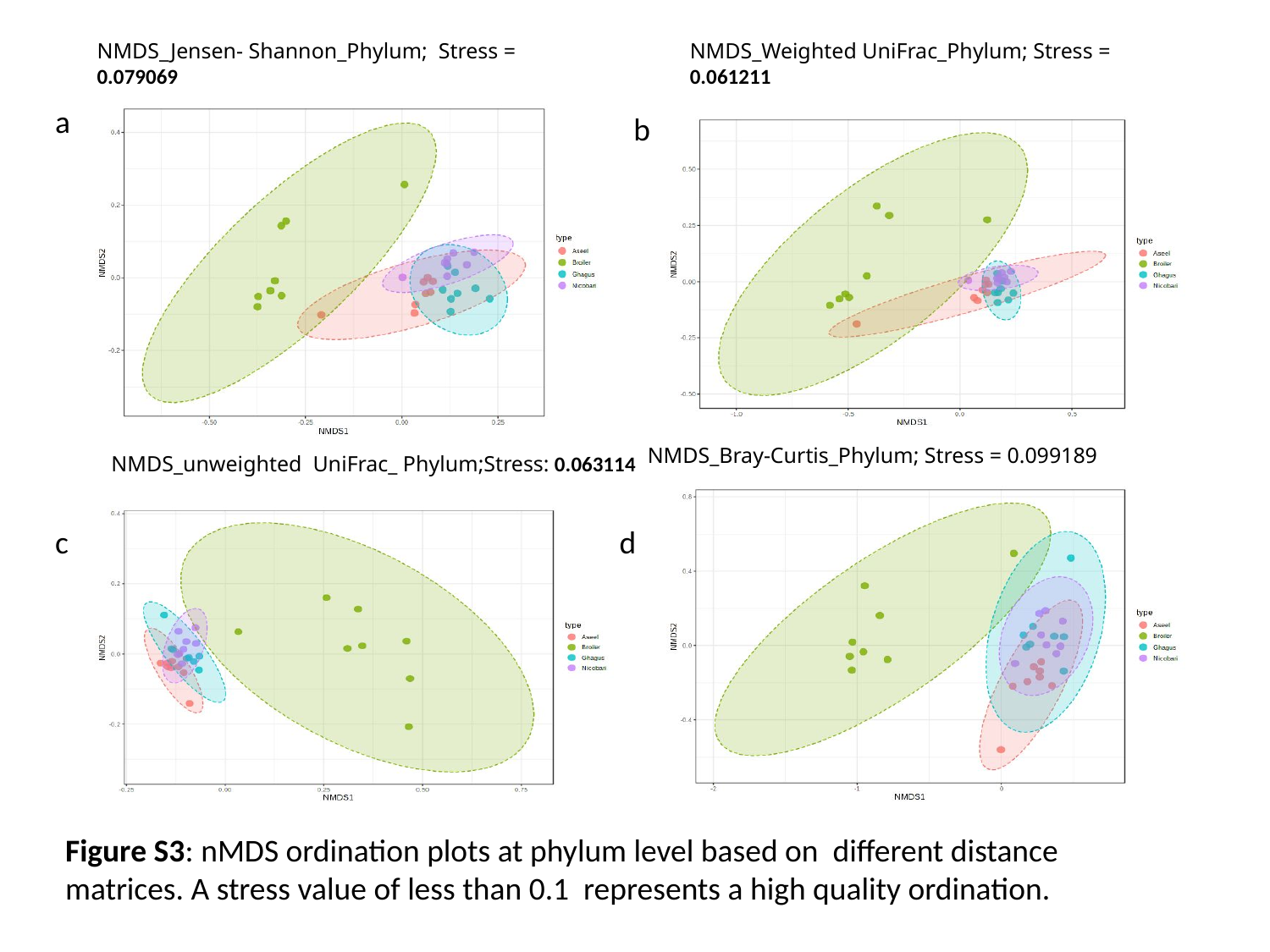

NMDS_Jensen- Shannon_Phylum; Stress = 0.079069
NMDS_Weighted UniFrac_Phylum; Stress = 0.061211
a
b
NMDS_Bray-Curtis_Phylum; Stress = 0.099189
NMDS_unweighted UniFrac_ Phylum;Stress: 0.063114
c
d
Figure S3: nMDS ordination plots at phylum level based on different distance matrices. A stress value of less than 0.1 represents a high quality ordination.
