# Supplementary material for: Gut Microbial Composition Differs Extensively among Indian Native Chicken Breeds Originated in Different Geographical Locations and a Commercial Broiler Line, but Breed-Specific, as Well as Across-Breed Core Microbiomes, Are Found
Source: Microorganisms. 2021 Feb 14;9(2):391. doi: 10.3390/microorganisms9020391 (PMC7918296; doi:10.3390/microorganisms9020391)
Supplement: Supplementary file 1 [file microorganisms-09-00391-s001.zip › Figure S5.pptx]

## Slide 1
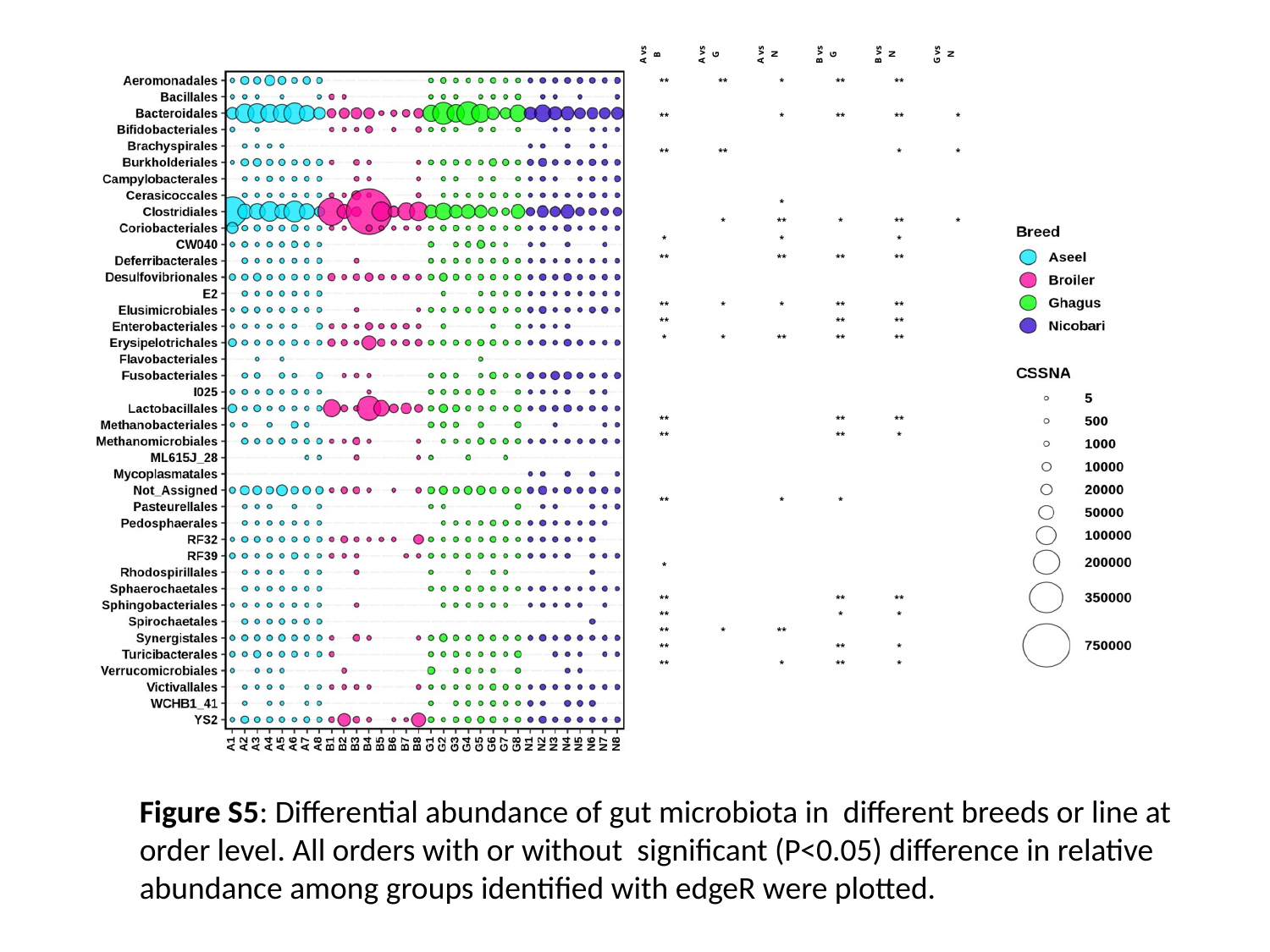

| A vs B | A vs G | A vs N | B vs G | B vs N | G vs N |
| --- | --- | --- | --- | --- | --- |
| \*\* | \*\* | \* | \*\* | \*\* | |
| | | | | | |
| \*\* | | \* | \*\* | \*\* | \* |
| | | | | | |
| \*\* | \*\* | | | \* | \* |
| | | | | | |
| | | | | | |
| | | \* | | | |
| | \* | \*\* | \* | \*\* | \* |
| \* | | \* | | \* | |
| \*\* | | \*\* | \*\* | \*\* | |
| | | | | | |
| | | | | | |
| \*\* | \* | \* | \*\* | \*\* | |
| \*\* | | | \*\* | \*\* | |
| \* | \* | \*\* | \*\* | \*\* | |
| | | | | | |
| | | | | | |
| | | | | | |
| | | | | | |
| \*\* | | | \*\* | \*\* | |
| \*\* | | | \*\* | \* | |
| | | | | | |
| | | | | | |
| | | | | | |
| \*\* | | \* | \* | | |
| | | | | | |
| | | | | | |
| | | | | | |
| \* | | | | | |
| | | | | | |
| \*\* | | | \*\* | \*\* | |
| \*\* | | | \* | \* | |
| \*\* | \* | \*\* | | | |
| \*\* | | | \*\* | \* | |
| \*\* | | \* | \*\* | \* | |
| | | | | | |
| | | | | | |
| | | | | | |
| | | | | | |
Figure S5: Differential abundance of gut microbiota in different breeds or line at order level. All orders with or without significant (P<0.05) difference in relative abundance among groups identified with edgeR were plotted.
