# Supplementary material for: Gut Microbial Composition Differs Extensively among Indian Native Chicken Breeds Originated in Different Geographical Locations and a Commercial Broiler Line, but Breed-Specific, as Well as Across-Breed Core Microbiomes, Are Found
Source: Microorganisms. 2021 Feb 14;9(2):391. doi: 10.3390/microorganisms9020391 (PMC7918296; doi:10.3390/microorganisms9020391)
Supplement: Supplementary file 1 [file microorganisms-09-00391-s001.zip › Figure S7.pptx]

## Slide 1
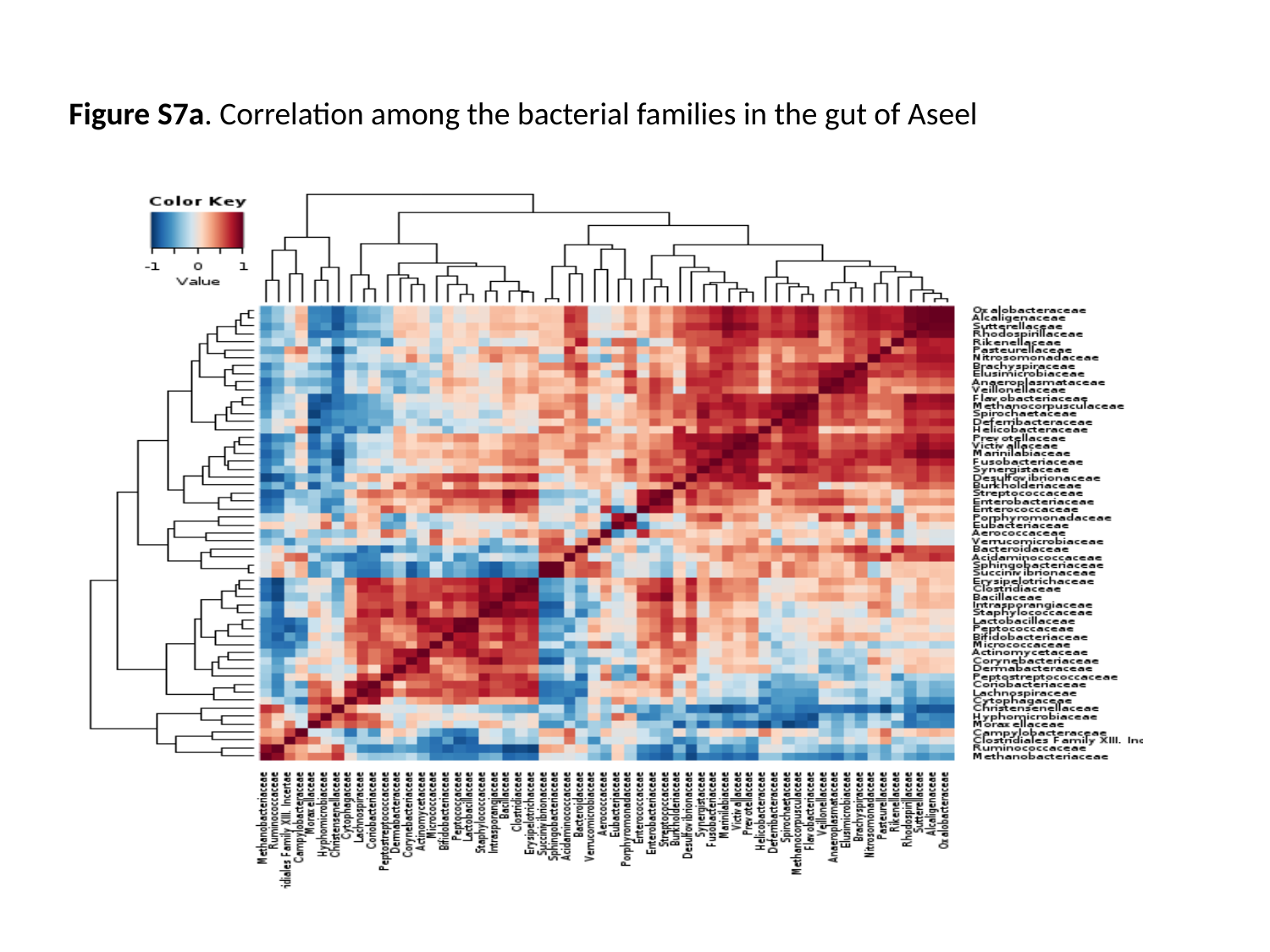

Figure S7a. Correlation among the bacterial families in the gut of Aseel

## Slide 2
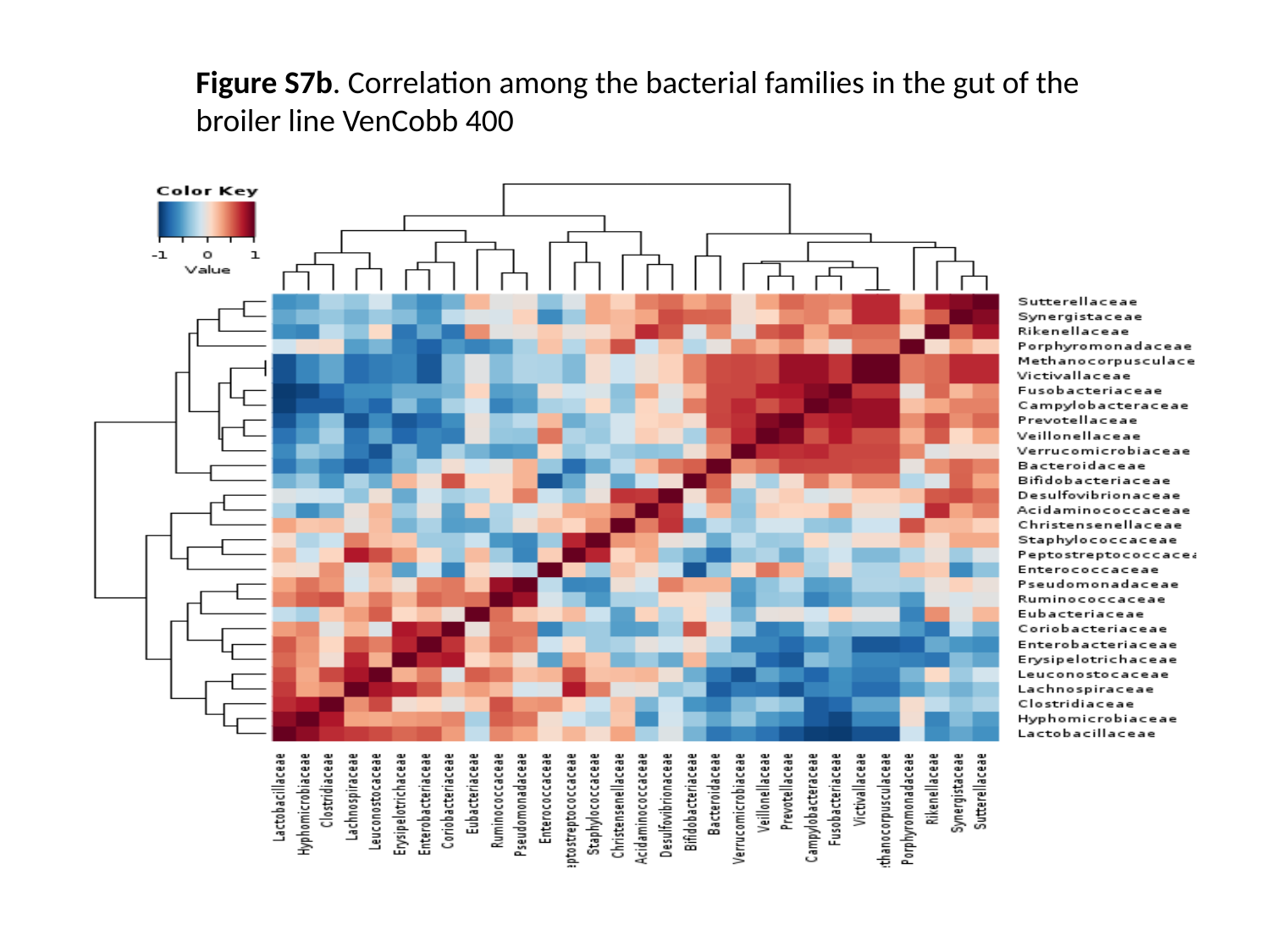

Figure S7b. Correlation among the bacterial families in the gut of the broiler line VenCobb 400

## Slide 3
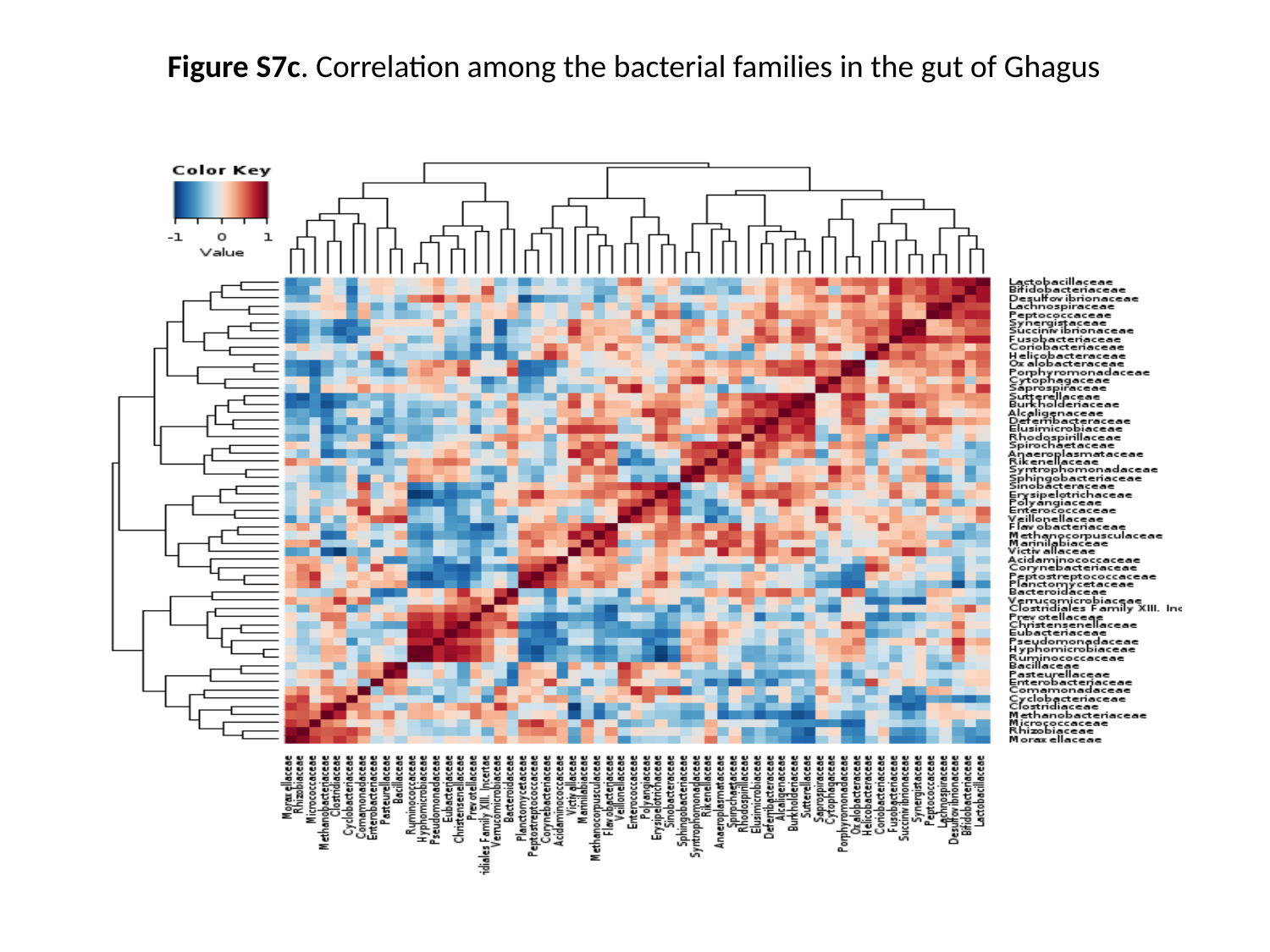

Figure S7c. Correlation among the bacterial families in the gut of Ghagus

## Slide 4
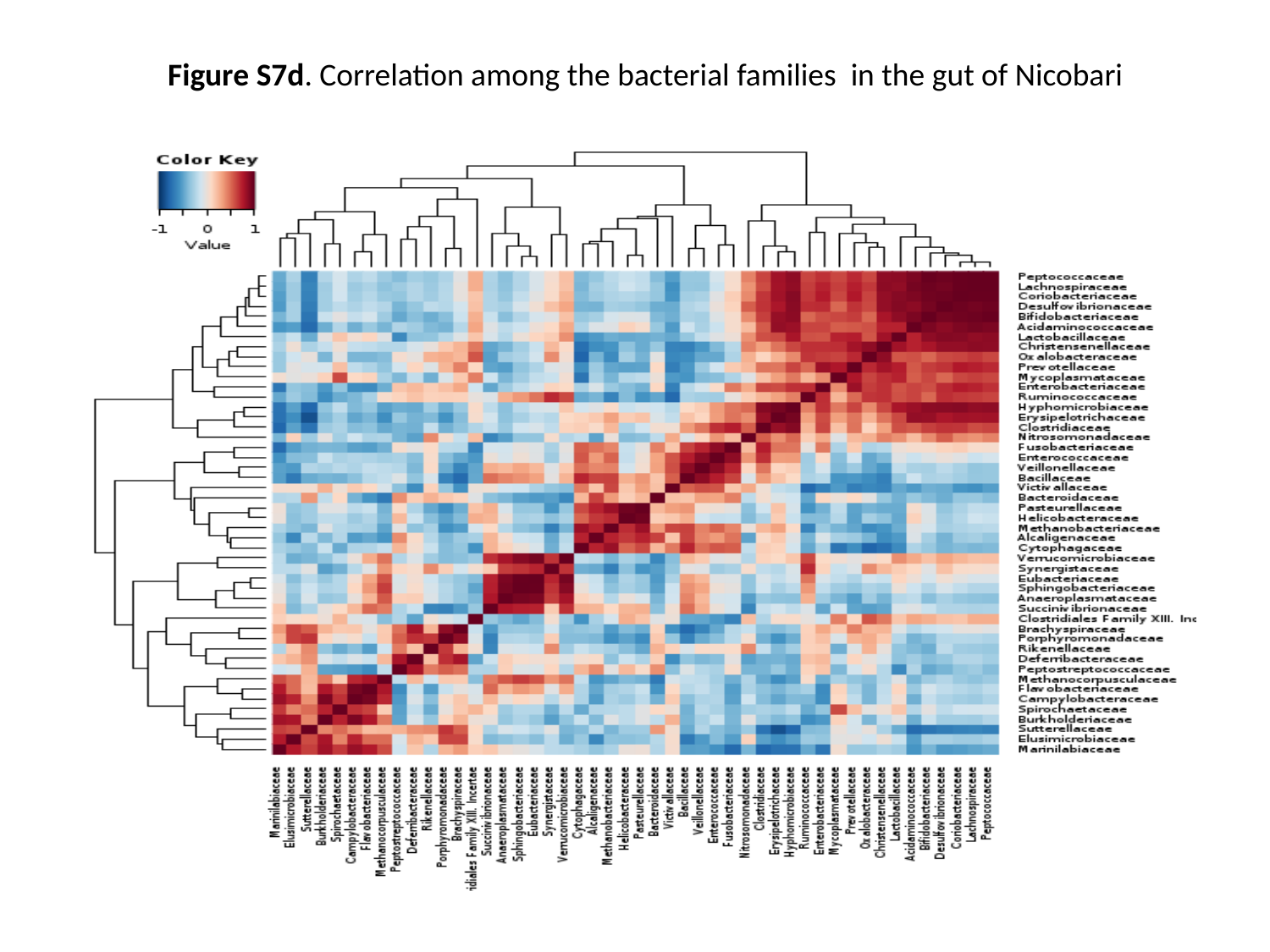

Figure S7d. Correlation among the bacterial families in the gut of Nicobari
