# Supplementary material for: Gut Microbial Composition Differs Extensively among Indian Native Chicken Breeds Originated in Different Geographical Locations and a Commercial Broiler Line, but Breed-Specific, as Well as Across-Breed Core Microbiomes, Are Found
Source: Microorganisms. 2021 Feb 14;9(2):391. doi: 10.3390/microorganisms9020391 (PMC7918296; doi:10.3390/microorganisms9020391)
Supplement: Supplementary file 1 [file microorganisms-09-00391-s001.zip › Table S1.docx]

**Table S1a Ingredient and nutrient composition of diets offered to the native chickens**

|  | Chicks (d 1-8 W) | Grower (9-17W) | Male breeder (17W onward) |
| --- | --- | --- | --- |
| Ingredient composition (g/kg) | | | |
| Maize | 623.6 | 562.0 | 659.5 |
| Soy bean meal | 254.0 | 240.9 | 150.0 |
| Deoiled rice bran | 78.18 | 153.0 | 147.6 |
| Stone grit | 15.78 | 18.6 | 21.8 |
| Dicalcium phosphate | 19.0 | 16.6 | 12.1 |
| DL-methionine | 1.28 | 1.10 | 0 |
| L-Lysine Hcl | 0.40 | 0 | 0 |
| Common salt | 3.5 | 3.5 | 4.0 |
| Trace mineral mix ^1^ | 1.0 | 1.0 | 1.0 |
| AB2D3K ^2^ | 0.15 | 0.15 | 0 |
| B complex vitamin ^3^ | 0.15 | 0.15 | 0 |
| Breeders vitamin premix | 0 | 0 | 1.0 |
| Choline chloride | 1.0 | 1.0 | 1.0 |
| Toxin binder(Bentonite) | 1.0 | 1.0 | 1.0 |
| Sodium bicarbonate | 1.0 | 1.0 | 1.0 |
| Nutrient composition (%) | | | |
| Metabolizable energy ^4^ (kcal/kg) | 2880 | 2810 | 2870 |
| Protein^5^ | 17.94 | 17.77 | 14.02 |
| d Lysine^4^ | 0.89 | 0.85 | 1.06 |
| d Methionine^4^ | 0.41 | 0.82 | 0.56 |
| d TSAA^4^ | 0.65 | 1.02 | 0.83 |
| d Threonine^4^ | 0.61 | 0.60 | 0.47 |
| d Tryptophan^4^ | 0.20 | 0.20 | 0.15 |
| Calcium^5^ | 1.11 | 1.16 | 1.16 |
| Total phosphorus^5^ | 0.83 | 0.87 | 0.74 |
| Available phosphorus^4^ | 0.50 | 0.46 | 0.37 |
| Na^4^ | 0.17 | 0.17 | 0.19 |
| Cl^4^ | 0.25 | 0.25 | 0.28 |

1 composition (g / kg): Mn: 100; Zn: 80; Cu: 15; Fe: 90; Se: 0.3; I: 2.0

2 composition (per g): A: 82500IU; B2:50mg; D3:12000IU; K: 12000 IU or 10 mg

3 composition (mg/per g):B1:4; B6:8; B12:0.04; E: 40; Calcium D pantothenate: 40; Niacin: 60; biotin: 0.2

4 calculated

5 Analysed

W, week

**Table S1b Ingredient and nutrient composition of diets offered to the broiler chickens**

|  | Pre-Starter (D 1-14) | Starter (D 15 to 28) | Finisher (D 29 onward) |
| --- | --- | --- | --- |
| Ingredient composition (g/kg) | | | |
| Maize | 539.8 | 556.1 | 582.0 |
| Soy bean meal | 387.7 | 356.4 | 310.5 |
| Stone grit | 24.5 | 10.6 | 10.7 |
| Fat (vegetable oil) | 19.0 | 42.9 | 63.4 |
| Dicalcium phosphate | 15.2 | 18.6 | 16.0 |
| DL-methionine | 3.05 | 2.65 | 2.67 |
| L-Lysine Hcl | 1.35 | 0.750 | 1.12 |
| L-Threonine | 0.653 | 0.260 | 0.39 |
| Common salt | 4.0 | 4.1 | 4.0 |
| Trace mineral mix ^1^ | 1.225 | 2.0 | 2.5 |
| AB2D3K ^2^ | 0.204 | 0.360 | 0.375 |
| B complex vitamin ^3^ | 0.204 | 0.360 | 0.375 |
| Choline chloride | 1.02 | 2.0 | 2.5 |
| Toxin binder(Bentonite) | 1.02 | 2.0 | 2.5 |
| Sodium bicarbonate | 1.02 | 1.0 | 1.0 |
| Nutrient composition (%) | | | |
| Metabolizable energy ^4^ (kcal/kg) | 2950 | 3100 | 3250 |
| Protein^5^ | 22.93 | 21.4 | 19.5 |
| d Lysine^4^ | 1.28 | 1.15 | 1.06 |
| d Methionine^4^ | 0.64 | 0.58 | 0.56 |
| d TSAA^4^ | 0.95 | 0.87 | 0.83 |
| d Threonine^4^ | 0.86 | 0.77 | 0.71 |
| d Tryptophan^4^ | 0.27 | 0.25 | 0.23 |
| Calcium^5^ | 1.39 | 0.93 | 0.88 |
| Total phosphorus^5^ | 0.71 | 0.76 | 0.68 |
| Available phosphorus^4^ | 0.44 | 0.50 | 0.44 |
| Na | 0.18 | 0.19 | 0.18 |
| Cl | 0.27 | 0.28 | 0.27 |

1 composition (g / kg): Mn: 100; Zn: 80; Cu: 15; Fe: 90; Se: 0.3; I: 2.0

2 composition (per g): A: 82500IU; B2:50mg; D3:12000IU; K: 12000 IU or 10 mg

3 composition (mg/per g):B1:4; B6:8; B12:0.04; E: 40; Calcium D pantothenate: 40; Niacin: 60; biotin: 0.2

4 calculated

5 Analysed
