# Supplementary material for: Gut Microbial Composition Differs Extensively among Indian Native Chicken Breeds Originated in Different Geographical Locations and a Commercial Broiler Line, but Breed-Specific, as Well as Across-Breed Core Microbiomes, Are Found
Source: Microorganisms. 2021 Feb 14;9(2):391. doi: 10.3390/microorganisms9020391 (PMC7918296; doi:10.3390/microorganisms9020391)
Supplement: Supplementary file 1 [file microorganisms-09-00391-s001.zip › Table S2.docx]

**Table S2: Statistical analysis of beta diversity measures for comparing microbial community composition between different breeds (PERMANOVA and PERMDISP results along with PCoA/NMDS parameters).**

|  |  |  | | | PCoA | | NMDS |  |
| --- | --- | --- | --- | --- | --- | --- | --- | --- |
| Taxonomic level | Diversity metric | PERMANOVA  Pseudo-F R^2^ P-value | | | % variation explained at  PC1 | % variation explained at  PC2 | Stress | PERMDISP-P-value |
| OTU | Bray-Curtis | 6.429 | 0.407 | <0.001 | 27.8 | 13.9 | 0.0862 | 0.855 |
|  | Jaccard | 3.987 | 0.299 | <0.001 | 18.2 | 11.0 | 0.0862 | 0.927 |
|  | Jensen-Shannon | 11.56 | 0.553 | <0.001 | 43.0 | 15.1 | 0.0914 | 0.788 |
|  | Weighted Unifrac | 8.378 | 0.473 | <0.001 | 48.3 | 10.3 | 0.0623 | 0.079 |
|  | Unweighted Unifrac | 5.200 | 0.358 | <0.001 | 23.3 | 9.7 | 0.0690 | 0.072 |
| Phylum | Bray-Curtis | 9.687 | 0.509 | <0.001 | 42.4 | 15.6 | 0.0991 | 0.546 |
|  | Jaccard | 6.112 | 0.395 | <0.001 | 28.5 | 14.4 | 0.0991 | 0.602 |
|  | Jensen-Shannon | 18.54 | 0.665 | <0.001 | 69.7 | 12.1 | 0.0791 | 0.584 |
|  | Weighted Unifrac | 10.40 | 0.527 | <0.001 | 58.4 | 10.1 | 0.0612 | 0.163 |
|  | Unweighted Unifrac | 9.146 | 0.494 | <0.001 | 44.6 | 8.5 | 0.0631 | <0.001 |
